# Reliable and Valid Coding of Thin Slices of Video Footage: Applicability to the Assessment of Mother-Child Interactions

**DOI:** 10.1007/s10862-017-9630-x

**Published:** 2017-11-04

**Authors:** Nicole Hirschmann, Ursula Kastner-Koller, Pia Deimann, Manuel Schmelzer, Jakob Pietschnig

**Affiliations:** Faculty of Psychology, Department of Applied Psychology: Health, Development, Enhancement, and Intervention, University of Vienna, Liebiggasse 5, A-1010 Wien, Austria

**Keywords:** Thin-slice sampling, Mother-child interaction, Behavioral observation, INTAKT, Reliability, Validity

## Abstract

When using behavioral-observation methods for coding video footage, it is unknown how much time of an interaction needs to be coded to gain results that are representative for the behavior of interest. The current study examined this problem using the INTAKT, a standardized observational measure for assessing the quality of mother-child interactions. Results from coding only 10 min of each video (i.e., thin slices) were compared with results from coding the remaining parts (averaging about 40 min) of the interaction. Inter-rater agreement for the short versions taken from the beginning or the middle, but not the end of the interactions indicated satisfactory observer accuracy. Coding results did not differ between short and long video sequences, when sequences were taken from the middle of the interactions. Importantly, characteristic differences between different interactive situations were equally well represented in the short and long video sequences. Therefore, our results show that coding only 10 min of an interaction is as reliable and valid as coding full-length videos, if those short sequences are taken from the middle of an interaction. Our findings support the idea that for every method, it is necessary to individually determine the window duration that is long enough to gain results that are reliable and valid.

## Introduction

The behavioral observation of interactions between mothers and their children can be an important part of the psychological assessment process. Oftentimes, video recordings are used as helpful tools for observing interactions, but their coding is frequently more time-consuming than desirable in practice. Therefore, coding of thin slices (i.e., short segments of observational video recordings) may prove beneficial to increase the usefulness of observational methods. However, even though it is common practice to use only a small portion of the video in order to minimize the necessary effort, there does not seem to be unequivocal empirical evidence that supports such an approach. Rather, studies that have examined this topic in more detail yielded often quite different conclusions.

A meta-analysis by Ambady and Rosenthal (1992) suggests that objective outcomes can be predicted from short behavioral observations. To refer to those short observations, they coined the term “thin slices”. They investigated studies from the areas of clinical and social psychology that had used slices of no more than five minutes of behavior and found an effect size for the accuracy of predictions of *r* = .39. They argued that other studies that did not use behavioral observation but had used different methods (e.g., self-reports, ratings) to predict similar criterion variables found similar effect sizes. Moreover, comparing studies that used shorter or longer sequences (from less than 30 s to 5 min) showed that duration of behavioral observation did not have an impact on observed effects. However, they did not find or use any studies that directly compared results from coding longer segments with coding shorter segments of the same situation.

More recently, James et al. (2012) focused on thin-slice sampling in the context of observing mother-child interactions. In a review of studies that used micro-analytic methods to code interaction behavior between parents and children, they found that 18 out of 38 published studies had used observation windows of 5 min or less for coding. Only 12 of those studies gave a justification for the duration of the observation window. Most of them argued that they either followed (their own) previous research or (e.g., Beebe et al. 2010) cited the meta-analysis by Ambady and Rosenthal (1992) in support of their approach.

In their own study, James et al. (2012) compared results from an 18 min parent-child interaction with results from coding 3, 6, or 9 min of the same interaction. They found that coding short slices (3 min or 6 min) did not yield the same pattern of results as coding the entire session. Only results from the beginning 9 min were consistently non-discrepant from results of the entire session. They concluded that their data do not support the applicability of Ambady and Rosenthal’s (1992, 1993) conclusions.

Considering this conflicting evidence, it seems important to empirically test how much time needs to be coded, when using behavioral-observation methods. Therefore, when developing the behavioral-observation method INTAKT (Hirschmann et al. 2011; an instrument for assessing the quality of mother-child interactions; see below), the original approach was to code the full-length videos (i.e., the complete recording of the interaction between mother and child). Because this is very time-consuming, the current study aims at determining whether coding thin slices of mother-child interactions yields results that are as reliable (in terms of inter-rater agreement) and valid as assessments based on full-length video observations.

INTAKT is a behavioral observation method that assesses the quality of mother-child interactions on three dimensions: Maternal sensitivity, maternal feedback to the child, and maternal interactive style in joint-attention episodes. The three dimensions were chosen for the assessment of the quality of maternal interactive style, because – as detailed below – those components of maternal interactive behavior are known to exhibit an important influence on the development of a child.

Maternal sensitivity (defined as a mother’s ability to perceive and interpret the signals and communications that are implicit in her infant’s behavior accurately and to respond to them appropriately and promptly; Ainsworth et al. 1974) facilitates children’s development of a secure attachment to their mother (e.g., De Wolff and van IJzendoorn 1997). Moreover, higher maternal sensitivity is correlated with higher socio-emotional as well as cognitive skills (e.g., Raikes and Thompson 2008; Stams et al. 2002).

Maternal feedback to her child can be either positive, corrective, or negative (e.g., Kelley et al. 2000). While positive and corrective maternal feedback are, for example, related to children’s persistence in the face of difficulty, negative feedback has been shown to be associated with a more negative cognitive style in the child (Kelley et al. 2000; Mezulis et al. 2006).

During episodes of joint attention, children share an attentional focus with a social partner which has been shown to benefit them in at least two different ways: On the one hand, such situations facilitate children’s language learning (Dominey and Dodane 2004). On the other hand, spending more time in joint-attention situations is associated with a more favorable social development (Vaughan Van Hecke et al. 2007). To children older than 6 months, it is especially conducive if mothers follow their attentional focus rather than manipulating it (Saxon et al. 2000).

INTAKT assesses these three dimensions of maternal interactive quality during two different situations. At first, mother and child are working with crafting materials to color and embellish a copy of a black-and-white drawing of a house. Later, they are offered a box containing different toys, which they can use for free play. Whilst the first situation is more structured with mother and child working on a task to be accomplished, the latter is an unstructured situation in which mother and child are free to play as they wish. Thus, the two situations are expected to elicit different maternal behaviors.

On the one hand, in structured, goal-oriented situations that can be seen as guided-learning interactions (Grusec and Davidov 2010), mothers assume the role of a teacher (Pasiak and Menna 2015). They are, therefore, expected to be emotionally available and to facilitate the child’s management of the task (Edhborg et al. 2001). They will also likely provide more feedback and exhibit more teaching behaviors as well as more attention-directing behaviors than during free toy-play (Grusec and Davidov 2010; Mateus et al. 2013). On the other hand, in unstructured, free-play situations that can be seen as interactions characterized by reciprocity (Grusec and Davidov 2010), mothers assume the role of a playmate (Pasiak and Menna 2015). They are, therefore, expected to be playful and to enjoy the time with their child (Edhborg et al. 2001). They will also likely exhibit higher levels of shared positive affect and lower levels of joint attention than during more structured tasks (Mateus et al. 2013; Pasiak and Menna 2015).

Because the INTAKT has been shown to assess the quality of maternal interactive style reliably and validly (Hirschmann et al. 2011), it may be deemed to be a useful instrument for routine use in the psychological assessment process, when working with children and their parents. So far, though, videos of interactions between mothers and children that had been recorded for the development of INTAKT had an average length of about 50 min (first about 20 min of crafting, then about 30 min of free play). Therefore, coding all three dimensions may be more time-consuming than desirable in practice. Thus, the aim of the current study is to examine if, when using the behavioral observation system INTAKT, coding short parts of video sequences (i.e., 4 min of crafting and 6 min of free play), taken from either the beginning, the middle, or the end of the crafting and the free-play situation, yields comparable results to coding the full-length video.

## Method

### Participants

This study used data from 80 mother-child dyads who had participated in various studies concerning the coding system INTAKT. There were 46 girls and 34 boys in the sample. Their age ranged from 3.0 to 5.9 years (mean age = 4.3 years; *SD* = 0.8 years). Maternal age ranged from 24 to 50 years (mean age = 34.2 years; *SD* = 5.8 years). All mothers gave informed, written consent for the videos to be used for research purposes.

### Selection of Videos

Out of 120 videos that had been videotaped and coded in the process of developing INTAKT, we selected includable videos according to the following three inclusion criteria: First, videos for which sufficient inter-rater reliability could not be assured were excluded. For each coder, inter-rater reliability of videos that were eligible for this study was estimated by comparing his/her codings with those of a second coder who independently coded approximately 25% of the same videos. Only videos of coders who had obtained at least moderate inter-observer agreement were included (i.e., Cohen κ > .4; see Landis and Koch 1977), thus reducing the number of videos by 26. All of the eligible videos had been coded by trained coders. Training was conducted by the developers of INTAKT and consisted of an introduction into behavioral observation as a data collection method and an introduction into important aspects of mother-child interactions, as well as an elaboration on the INTAKT coding dimensions. Each category was studied via its description in the INTAKT manual as well as via various video-recorded examples of mother-child interactions. After training, each coder coded a trial video. Results were compared to a completed coding form from the test developers. Feedback was then given to coders, including a review and clarification of coding errors. Coding of additional trial videos followed, if deemed necessary.

Second, videos in which either the structured or the unstructured interaction lasted too short (i.e., either less than 10 or 14 min for crafting or free play, respectively) were excluded. Each video consisted of a structured situation in which mother and child could work with craft materials and an unstructured free-play situation. Mother and child were free to decide how much time they wished to spend on each task, in order to avoid frustration in the young children by taking away crafting materials before their work was finished. Therefore, videos differed in length. On average, working with the craft materials lasted for 20:40 min and the free-play situation lasted for 28:20 min (roughly a 2:3 ratio). In a few videos, one or the other situation lasted for only a short time. Therefore, it was necessary to determine a minimum length of each situation that still allowed for a meaningful shortening of the sequence (because shortening the videos was the purpose of the study). Those minimums were set at 10 min for the crafting situation and 14 min for the free-play situation (representing a similar ratio as in the original durations of videos). According to those criteria, another six videos had to be excluded.

Third, after particularly short videos had been excluded in our second step, particularly long videos were excluded as well. This concerned eight videos for which the duration of the crafting and/or the free-play situation deviated extremely from the average. For each video, differences between the duration of each situation and the average duration were squared and summed up to determine which videos showed a particularly large deviation from the norm. According to this criterion, 10% of videos which differed the most from the average were excluded. Therefore, after this final selection 80 videos were included in the present study. These videos had an average length of 46:56 min (19:38 min of crafting and 27:18 min of free play).

### Recording and Editing

For video recording interactions between mothers and their children, crafting materials (e.g., colored pens, colored papers, scissors, and glue) were prepared on a table and a box containing various play materials (e.g., puppets, cars, small furniture) was placed aside. Mother and child were told, “Look, I have got a crafts project for you! Would you [instructor looks at child] like to turn this boring house [instructor points to a copy of a black-and-white drawing of a house] into a beautiful and colorful house? Your Mum can help you and you can use all the materials you can find on the table. The house is done as soon as [child’s name] says so. When you are done with the house there [instructor points to box with play materials] is something to play with, for you and your Mum.” They were allowed to take a break if desired. Following the recording of the interaction, each camera file was converted into WMV format with 24 frames per second.

### Behavioral Coding

Each video was coded with the behavioral coding system INTAKT (Hirschmann et al. 2011), which assesses maternal interactive style on three dimensions: *Sensitivity*, *Feedback*, and *Joint Attention*. Each dimension is coded separately, thus making it necessary to watch the video at least three times. *Sensitivity* is rated on a 7-point Likert-typed scale, ranging from *very low sensitivity* (1) to *very high sensitivity* (7). Every other step of the rating scale is precisely verbally anchored, thus giving exact descriptions of corresponding maternal behaviors. Descriptions, for example, focus on whether the mother notices the child’s signals and reacts promptly and appropriately to them, whether she can adopt the child’s viewpoint, and whether her language is appropriate for the child’s developmental status. For coding, the video is stopped after two minutes, watched again if necessary, and then maternal behavior during this interval is rated according to the scale. This procedure is repeated every two minutes until the end of the video.

*Feedback* is a classification system comprising four categories: *positive feedback* (e.g., mother praises the child for having had a good idea), *corrective feedback* (e.g., mother uses a friendly voice to tell the child how to better hold the scissors), *negative feedback* (e.g., mother tells the child that his drawing is ugly), and *no feedback* (mother gives no feedback to the child). *Joint Attention* is a classification system comprising six categories: *active maintenance* (e.g., mother is taking part in the child’s role-play with dolls, thereby following the child’s ideas for the game), *verbal maintenance* (e.g., mother acknowledges that the child has now colored the house and asks what he is going to do next), *passive maintenance* (e.g., mother silently watches the child draw), *attention manipulation* (e.g., mother tells the child that he must now use a blue instead of a red pen), *attention switching* (e.g., mother tells the child that they cannot play with dolls anymore and must now play with cars instead), and *no joint attention* (e.g., mother looks at her mobile phone while the child is painting the house). For both classification systems, categories are mutually exclusive and exhaustive. Therefore, within each classification system one of the categories is coded for every moment of the video (event-sampling method; see, for instance, Bakeman and Quera 2011). The software INTERACT (Mangold 2011) was used to accomplish coding of all three dimensions.

Coding of all 80 videos was shared by six different coders, who coded 4, 5, 12, 16, 19, and 24 videos, respectively. Twenty-two (27.5%) of those videos were independently coded by a second coder to obtain estimates for inter-rater agreement. Coder 1 additionally coded two videos that had already been coded by Coder 2, and Coder 2 additionally coded two videos that had already been coded by Coder 1. Furthermore, a seventh coder coded 18 videos that had already been coded (five from Coder 3, 2 from Coder 4, 5 from Coder 5, and 6 from Coder 6). Inter-rater agreement for maternal *Sensitivity* reached an ICC = .563, 95% CI [.502, .618] for single ratings and an ICC = .654, 95% CI [.337, .839] for the average ratings per video. Inter-rater agreement for *Feedback* reached κ = .59/.63 and for *Joint Attention*, it was at κ = .74/.77 (see next section for details on how reliability coefficients were calculated).

### Data Extraction and Data Analyses

Means for maternal-sensitivity ratings were calculated using SPSS. Durations and frequencies of all categories of *Feedback* and *Joint Attention* were calculated with INTERACT. For every video, this was done for the whole video, for the shortened versions of the video (4 min crafting and 6 min free play), and for the respective rests of the video. Results from INTERACT were transferred into Microsoft Excel and SPSS, where all further calculations were accomplished.

Inter-rater reliability for maternal *Sensitivity* was examined using SPSS. Because *Sensitivity* is assessed on a rating scale and videos were coded by different coders, intra-class correlation coefficients (ICC; one-way random effects model) were calculated. Inter-rater agreement for *Feedback* and *Joint Attention* were examined using the Generalized Sequential Querier (GSEQ, Version 5.1). Because *Feedback* and *Joint Attention* were coded with an event-sampling method, *time-unit kappa with tolerance* was used as an agreement measure. To this end, the stream of events is divided into equal units (1 s intervals in our case). For each time unit, it is observed whether the other coder decided for the same category within a specified time window of tolerance (plus/minus 3 s in our case). GSEQ then reports two kappa values for each calculation, one for each coder as the first one (Bakeman and Quera 2011, p. 78).

All videos that were recorded for coding with INTAKT consisted of two different situations. Initially, mothers and children were instructed to work together to craft a house. Subsequently, they were given a box of play materials that they could use for engaging in free play. Most mother-child dyads spent less time on the crafting situation than on the free-play situation (a 2:3 ratio was typically observed). In all videos that were used for analysis, the crafting situation was at least 10 min long and the free-play situation lasted at least 14 min (see “Selection of Videos” section above). As maternal sensitivity is coded in 2-min intervals, it was reasonable to choose an even number of minutes for the shortened version. Therefore, it was decided to use 4 min of the crafting situation and 6 min of the free-play situation to represent the shortened version of the videos (yielding once more a 2:3 ratio). Those 10 min constitute about 1/5 of the average length of full-length videos, which would mean that coding could be reduced by about 4/5 of the time.

All analyses were done for three different time points. First, 4 min of the crafting situation and 6 min of the free-play situation were taken starting from one minute after crafting and one minute after free play started. Second, the length of the crafting situation was divided in half and a 4 min interval that started 2 min before and ended 2 min after that point was taken. Similarly, for the free-play situation, a 6 min interval that started 3 min before and ended 3 min after half of the free-play situation was taken. Third, 4 min of the crafting situation and 6 min of the free-play situation were taken from one minute before crafting and one minute before free play ended.

To examine whether coding shorter segments is as reliable and valid as coding the full-length video, three analyses were conducted. First, inter-rater reliabilities were examined. To this end, observer-agreement indices for the shortened versions were compared with observer-agreement indices for the full-length videos. Second, to investigate whether results from the shortened versions are comparable with results from the long versions, results from the shortened version were compared with results from the respective rests of the videos. We consciously chose not to compare the sequences with the full-length videos to provide a conservative estimate (i.e., to avoid overestimating agreement). Third, for further validation we assessed typical differences between the crafting situation and the free-play situation in the full-length videos and examined if the same differences appeared in the shortened versions.

## Results

### Inter-Rater Agreement

Maternal *Sensitivity* was coded for every two minutes of a video. For analyses, those ratings can be averaged per video to yield a value that represents the mean level of maternal *Sensitivity* for a given interaction. Thus, intra-class correlations for maternal *Sensitivity* can be calculated either for the single ratings or for the averaged ratings per video. As can be seen in Table 1, inter-rater agreements did not differ significantly from each other when the full-length video was being coded or when only a short sequence was coded. This was true regardless of that sequence having been taken from the beginning, the middle, or the end of each situation.

**Table 1:** Inter-rater agreement for shortened versions and full-length videos

INTAKT category	Full-length videos	Shortened versions – beginning	Shortened versions – middle	Shortened versions – end
*Sensitivity*				
Single ratings	ICC = .56, 95% CI [.502, .618]	ICC = .58, 95% CI [.467, .680]	ICC = .57, 95% CI [.451, .670]	ICC = .61, 95% CI [.492, .707]
Averaged ratings	ICC = .65, 95% CI [.337, .839]	ICC = .64, 95% CI [.309, .830]	ICC = .62, 95% CI [.279, .827]	ICC = .67, 95% CI [.349, .850]
*Feedback*	κ = .62, 95% CI [.60, .64]	κ = .61, 95% CI [.56, .66]	κ = .73, 95% CI [.68, .77]	κ = .53, 95% CI [.49, .57]
	κ = .60, 95% CI [.58, .62]	κ = .57, 95% CI [.52, .63]	κ = .70, 95% CI [.65, .74]	κ = .52, 95% CI [.48, .57]
*Joint Attention*	κ = .74, 95% CI [.74, .75]	κ = .70, 95% CI [.68, .71]	κ = .68, 95% CI [.66, .69]	κ = .77, 95% CI [.76, .78]
	κ = .78, 95% CI [.77, .78]	κ = .74, 95% CI [.73, .75]	κ = .71, 95% CI [.70, .72]	κ = .79, 95% CI [.78, .81]

Regarding *Feedback*, *time-unit kappas with tolerance* showed no significant difference between full-length videos and a shortened sequence taken from the beginning of each situation. Kappas were significantly higher for a shortened sequence that was taken from the middle, and significantly lower for a shortened sequence that was taken from the end of each situation than for full-length videos.

For *Joint Attention*, codings of the shortened versions that had been taken from the beginning or the middle of each situation yielded significantly lower kappa values than codings of the full-length videos, whereas codings of a shortened sequence that had been taken from the end of each situation yielded significantly higher kappa values (see Table 1).

### Comparison between the Shortened Version and the Remaining Interaction

In order to examine whether coding shorter segments of the interaction is representative of coding the whole interaction, results from the shortened versions were compared with results from the rest of the interaction. At first, possible differences between the shortened versions and the rest of the interactions were examined (see Table 2). Then, correlations between the shortened versions and the rest of the interactions were analyzed (see Table 3).

**Table 2:** Comparison between shortened versions and remaining parts of videos

	Crafting					Free play				
INTAKT category	M (SD) – short	M (SD) – rest	*t*	*p*	*d*	M (SD) – short	M (SD) – rest	*t*	*p*	*d*
Beginning										
*Sensitivity*	5.60 (0.98)	5.58 (0.98)	0.48	.631	0.054	5.76 (0.93)	5.77 (0.92)	−0.29	.774	−0.032
*Feedback*										
*positive feedback*	2.28 (2.77)	2.03 (1.71)	0.94	.349	0.105	0.67 (1.58)	0.66 (1.15)	0.10	.917	0.011
*corrective feedback*	2.19 (3.39)	1.81 (2.10)	1.01	.318	0.113	0.83 (1.50)	0.63 (1.00)	1.18	.240	0.132
*negative feedback*	0.47 (1.39)	0.47 (0.68)	0.01	.988	<0.001	0.19 (0.51)	0.27 (0.53)	−1.08	.284	−0.121
*no feedback*	94.35 (5.31)	95.04 (3.39)	−1.28	.205	−0.143	97.74 (2.57)	97.41 (2.35)	1.00	.320	0.112
*Joint Attention*										
*active maintenance*	56.00 (26.98)	51.29 (20.95)	1.82	.072	0.203	74.76 (17.86)	68.13 (15.87)	3.40	.001	0.380
*verbal maintenance*	13.24 (9.70)	17.07 (11.63)	−3.09	.003	−0.345	10.26 (9.56)	13.05 (9.64)	−2.13	.036	−0.238
*passive maintenance*	22.64 (18.10)	25.12 (16.43)	−1.39	.168	−0.155	11.44 (9.06)	13.60 (9.30)	−2.33	.023	−0.261
*attention manipulation*	5.34 (6.90)	4.31 (4.43)	1.73	.088	0.193	2.19 (3.40)	2.02 (2.14)	0.55	.582	0.061
*attention switching*	–	0.06 (0.18)	–	–	–	0.11 (0.52)	0.24 (0.80)	−1.76	.082	−0.197
*no joint attention*	0.05 (0.20)	0.24 (0.53)	−3.31	.001	−0.370	0.45 (1.56)	0.68 (2.10)	−1.43	.157	−0.160
Middle										
*Sensitivity*	5.64 (1.04)	5.56 (0.97)	1.72	.090	0.192	5.78 (0.93)	5.78 (0.89)	−0.17	.867	<−0.001
*Feedback*										
*positive feedback*	2.20 (2.47)	1.92 (1.53)	1.42	.158	0.159	0.60 (0.98)	0.70 (1.44)	−0.54	.590	−0.060
*corrective feedback*	1.72 (2.56)	1.82 (2.12)	−0.35	.730	−0.039	0.52 (1.19)	0.74 (1.08)	−1.44	.155	−0.161
*negative feedback*	0.53 (0.84)	0.47 (0.80)	0.56	.578	0.063	0.30 (0.70)	0.22 (0.40)	0.87	.386	0.097
*no feedback*	95.55 (3.91)	95.79 (2.80)	−0.62	.535	−0.069	98.58 (1.80)	98.34 (1.89)	0.90	.370	0.101
*Joint Attention*										
*active maintenance*	55.47 (23.69)	53.09 (18.82)	1.32	.192	0.148	68.92 (19.71)	71.03 (13.31)	−1.21	.229	−0.135
*verbal maintenance*	16.17 (13.45)	16.88 (9.66)	−0.60	.550	−0.007	13.59 (11.18)	12.52 (7.69)	1.10	.274	0.123
*passive maintenance*	24.07 (18.35)	24.81 (15.20)	−0.58	.565	−0.065	14.26 (11.22)	13.43 (9.07)	0.76	.451	0.085
*attention manipulation*	4.10 (5.91)	4.96 (4.73)	−1.75	.085	−0.196	2.24 (2.88)	2.21 (2.41)	0.09	.928	0.010
*attention switching*	–	0.06 (0.17)	–	–	–	0.24 (1.09)	0.21 (0.73)	0.31	.756	0.035
*no joint attention*	0.19 (1.19)	0.20 (0.43)	−0.07	.948	−0.008	0.75 (2.56)	0.60 (1.77)	0.89	.376	0.100
End										
*Sensitivity*	5.56 (1.04)	5.58 (0.97)	−0.40	.688	−0.045	5.77 (0.99)	5.76 (0.91)	0.23	.818	0.026
*Feedback*										
*positive feedback*	1.84 (1.77)	2.11 (1.93)	−1.37	.174	−0.153	0.50 (1.01)	0.72 (1.45)	−1.19	.237	−0.133
*corrective feedback*	1.65 (2.86)	1.81 (1.99)	−0.48	.635	−0.054	0.81 (1.60)	0.65 (0.97)	0.94	.352	0.105
*negative feedback*	0.52 (1.07)	0.47 (0.84)	0.34	.731	0.038	0.25 (0.61)	0.24 (0.38)	0.20	.844	0.022
*no feedback*	96.00 (1.07)	95.61 (3.11)	0.94	.352	0.105	98.44 (1.94)	98.38 (1.85)	0.21	.833	0.023
*Joint Attention*										
*active maintenance*	50.65 (26.08)	54.29 (18.74)	−1.52	.133	−0.170	67.21 (20.15)	71.95 (14.11)	−2.21	.030	−0.247
*verbal maintenance*	16.62 (14.49)	16.76 (9.58)	−0.11	.912	−0.012	14.15 (12.62)	12.12 (8.18)	1.39	.168	0.155
*passive maintenance*	28.37 (20.18)	23.69 (14.65)	2.94	.004	0.329	15.94 (13.33)	12.75 (8.30)	2.73	.008	0.305
*attention manipulation*	4.01 (5.40)	5.05 (5.47)	−1.68	.096	−0.188	1.85 (3.03)	2.32 (2.43)	−1.45	.150	−0.162
*attention switching*	0.09 (0.46)	0.04 (0.13)	0.97	.335	0.108	0.31 (1.70)	0.18 (0.55)	0.81	.418	0.091
*no joint attention*	0.25 (0.90)	0.17 (0.40)	0.82	.413	0.092	0.53 (2.02)	0.69 (1.97)	−0.99	.327	−0.111

**Table 3:** Correlations between shortened versions and remaining parts of videos

	Crafting			Free play		
INTAKT category	Beginning	Middle	End	Beginning	Middle	End
*Sensitivity*	.88** (.92**)	.94** (.95**)	.92** (.94**)	.87** (.94**)	.94** (.96**)	.89** (.92**)
*Feedback*						
*positive feedback*	.55** (.79**)	.69** (.85**)	.54** (.70**)	.82** (.92**)	.10 (.30**)	.08 (.28*)
*corrective feedback*	.30** (.59**)	.36** (.61**)	.30** (.64**)	.30** (.62**)	.30** (.52**)	.37** (.68**)
*negative feedback*	.50** (.79**)	.44** (.61**)	.13 (.43**)	.12 (.48**)	.11 (.63**)	.58** (.77**)
*no feedback*	.44** (.72**)	.54** (.74**)	.39** (.55**)	.28* (.55**)	.15 (.28*)	.26* (.35**)
*Joint Attention*						
*active maintenance*	.56** (.79**)	.73** (.78**)	.58** (.70**)	.47** (.72**)	.62** (.74**)	.42** (.64**)
*verbal maintenance*	.47** (.64**)	.62** (.79**)	.64** (.79**)	.25* (.57**)	.63** (.79**)	.26* (.65**)
*passive maintenance*	.58** (.76**)	.78** (.87**)	.71** (.83**)	.59** (.76**)	.55** (.68**)	.62** (.81**)
*attention manipulation*	.64** (.84**)	.68** (.83**)	.48** (.64**)	.58** (.82**)	.50** (.74**)	.47** (.70**)
*attention switching*	–	–	−.05 (.60**)	.49** (.59**)	.61** (.81**)	.59** (.85**)
*no joint attention*	.34** (.45**)	.06 (.62**)	.28* (.65**)	.72** (.84**)	.80** (.91**)	.75** (.87**)

Note. Numbers in parentheses are correlations between shortened versions and full-length videos

^†^ *p* < .10. *p < .05. **p < .01

For mean maternal *Sensitivity* there was no difference between the shortened version of the crafting situation and the rest of the crafting situation. There was no difference regarding *Sensitivity* between the shortened free-play situation and the rest of the free-play situation either. For both situations, this was the case regardless of which point in time the sequence was taken from.

Regarding *Feedback,* no differences emerged for the three time points between the shortened versions and the remaining parts of the video for any category. Mere visual inspection of the results shows that mothers gave *no feedback* most of the time (between 94.35% and 98.58% of the time). If they gave feedback, it was more often *positive* or *corrective* than *negative feedback* (the latter only between 0.19% and 0.53% of the time) for all three time points.

Regarding *Joint Attention,* some differences emerged between the shortened versions that were taken from the beginning of the two situations and the respective remaining parts of the videos (see Table 2). In the shortened versions of the crafting situation, mothers used less *verbal maintenance* when working with their children, never *switched* the attentional focus of their children, and the category *no joint attention* was almost entirely unrepresented. In the shortened versions of the free-play situation, mothers used more *active maintenance*, less *verbal maintenance*, and less *passive maintenance*. Concerning the shortened versions that were taken from the middle of the two situations, there were no differences between them and the respective remaining parts of the videos. The category *attention switching* seems to have played only a minor role in crafting situations. Whilst it has been only used for 0.05% of time in any crafting sequence, it had not been used at all in middle slices of crafting. When sequences were taken from the end of the two situations, some differences emerged. In the crafting situation, mothers used more *passive maintenance* in the short version than in the remaining parts of the videos. In the free-play situation, mothers used less *active maintenance* and more *passive maintenance* in the short versions than in the remaining parts of the videos.

To assess whether mother-child dyads retained their relative position when short segments instead of long sequences were used, correlations between shortened versions and the remaining parts of the videos were examined (see Table 3). For *Sensitivity*, all correlations exhibited large values (*r* = .87 to *r* = .94). For *Feedback,* correlations exhibited medium to large values in the crafting situation (with the exception of *negative feedback* in the end slice) and small to large values in the free-play situation. Correlations for *Joint Attention* yielded predominantly medium-sized to large coefficients. Of note, two correlations could not be calculated, because *attention switching* did not occur in the thin slices taken from the beginning or middle of the crafting situation.

### Differences between Crafting and Free Play

Because all INTAKT videos consist of two different situations that are expected to elicit different maternal behaviors, it was of interest whether these differences would emerge in the shortened versions as well.

In the full-length videos, mean maternal *Sensitivity* was higher in the free-play situation than in the crafting situation. A similar result was observed for the shortened versions of the videos, regardless of which point in time the shortened version was taken from. Of note, the difference did not reach nominal significance when the sequence was taken from the middle of the two situations (all values are presented in Table 4).

**Table 4:** Comparison between crafting and free play in the full-length videos and in the shortened versions

INTAKT category	M (SD) – crafting	M (SD) – free play	*t*	*p*	*d*	M (SD) – crafting	M (SD) – free play	*t*	*p*	*d*
	full-length videos					shortened versions – beginning				
*Sensitivity*	5.57 (0.96)	5.76 (0.89)	−3.46	.001	−0.387	5.60 (0.98)	5.76 (0.93)	−2.11	.038	−0.236
*Feedback*										
*positive feedback*	2.01 (1.69)	0.65 (1.16)	6.60	<.001	0.738	2.28 (2.77)	0.67 (1.58)	4.60	<.001	0.514
*corrective feedback*	1.84 (1.93)	0.68 (0.94)	5.79	<.001	0.647	2.19 (3.39)	0.83 (1.50)	3.39	.001	0.379
*negative feedback*	0.48 (0.72)	0.24 (0.39)	3.24	.002	0.362	0.47 (1.39)	0.19 (0.51)	1.87	.065	0.209
*no feedback*	94.98 (3.33)	97.53 (1.95)	−6.55	<.001	−0.732	94.35 (5.31)	97.74 (2.57)	−5.43	<.001	−0.607
*Joint Attention*										
*active maintenance*	52.72 (20.56)	69.66 (14.23)	−7.62	<.001	−0.852	56.00 (26.98)	74.76 (17.86)	−5.76	<.001	−0.644
*verbal maintenance*	16.33 (9.80)	12.36 (7.69)	3.52	.001	0.394	13.24 (9.70)	10.26 (9.56)	2.03	.046	0.227
*passive maintenance*	24.00 (14.78)	13.12 (8.52)	7.69	<.001	0.860	22.64 (18.10)	11.44 (9.06)	5.33	<.001	0.596
*attention manipulation*	4.67 (4.68)	2.10 (2.18)	5.76	<.001	0.644	5.34 (6.90)	2.19 (3.40)	4.34	<.001	0.485
*attention switching*	0.05 (0.14)	0.20 (0.67)	−2.06	.043	−0.230	–	0.11 (0.52)	–	–	–
*no joint attention*	0.19 (0.40)	0.61 (1.82)	−2.15	.035	−0.240	0.05 (0.20)	0.45 (1.56)	−2.26	.027	−0.253
	shortened versions – middle					shortened versions – end				
*Sensitivity*	5.64 (1.04)	5.78 (0.93)	−1.80	.076	−0.201	5.56 (1.04)	5.77 (0.99)	−2.62	.011	−0.293
*Feedback*										
*positive feedback*	2.20 (2.47)	0.60 (0.98)	6.13	<.001	0.685	1.84 (1.77)	0.50 (1.01)	6.37	<.001	0.712
*corrective feedback*	1.72 (2.56)	0.52 (1.19)	4.35	<.001	0.486	1.65 (2.86)	0.81 (1.60)	2.46	.016	0.275
*negative feedback*	0.53 (0.84)	0.30 (0.70)	1.90	.062	0.212	0.52 (1.07)	0.25 (0.61)	1.94	.056	0.217
*no feedback*	95.55 (3.91)	98.58 (1.80)	−7.44	<.001	−0.832	96.00 (3.50)	98.44 (1.94)	−5.39	<.001	−0.603
*Joint Attention*										
*active maintenance*	55.47 (23.69)	68.92 (19.71)	−4.23	<.001	−0.483	50.65 (26.08)	67.21 (20.15)	−5.05	<.001	−0.565
*verbal maintenance*	16.17 (13.45)	13.59 (11.18)	1.43	.156	0.160	16.62 (14.49)	14.15 (12.62)	1.41	.163	0.158
*passive maintenance*	24.07 (18.35)	14.26 (11.22)	5.04	<.001	0.563	28.37 (20.18)	15.94 (13.33)	5.93	<.001	0.663
*attention manipulation*	4.10 (5.91)	2.24 (2.88)	3.25	.002	0.363	4.01 (5.40)	1.85 (3.03)	3.43	.001	0.383
*attention switching*	–	0.24 (1.09)	–	–	–	0.09 (0.46)	0.31 (1.70)	−1.13	.263	−0.126
*no joint attention*	0.19 (1.19)	0.75 (2.56)	−1.77	.081	−0.198	0.25 (0.90)	0.53 (2.02)	−1.18	.242	−0.132

Regarding *Feedback*, there were differences between the crafting situation and the free-play situation in all categories of the full-length videos. Mothers gave less feedback in the free-play situation. *Positive feedback*, *corrective feedback*, and *negative feedback* were more prevalent in the crafting situation. The same pattern emerged for the shortened versions, indicating that mothers gave more feedback in the crafting situation, especially more *positive* and more *corrective feedback*. There was also a trend for more *negative feedback* in the crafting situation, although nominal significance was not reached. The same pattern was found for all three time points of the thin slices.

Regarding *Joint Attention*, full-length videos exhibited differences between the crafting situation and the free-play situation in all categories. There were less *active maintenance*, more *verbal maintenance*, more *passive maintenance*, more *attention manipulation*, less *attention switching*, and fewer observations of *no joint attention* in the crafting situation. The same pattern emerged for the shortened versions when they were taken from the beginning of the two situations. However, *attention switching* was never observed during the shortened crafting situations. When the shortened version was taken from the middle of the two situations, the pattern was similar with the exception that there was no difference between crafting and free play for *verbal maintenance*, and that the difference for *no joint attention* did not reach nominal significance. When the sequences were taken from the end of the two situations, the pattern was only partly replicated. There was no difference between crafting and free play for *verbal maintenance*, *attention switching*, and *no joint attention* (see Table 4).

## Discussion

In all, we show that inter-rater reliability and validity are satisfactory, even when coding only short segments of video-recorded mother-child interactions by means of the behavioral-observation method INTAKT. Concerning inter-rater agreement, observer-agreement indices for *Sensitivity* (i.e., ICCs) show no significant differences between full-length videos or shortened versions. This finding is consistent for all three shortened versions from different time points and holds up for single ratings as well as for averaged ratings. Regarding *Feedback,* observer-agreement indices (i.e., Cohen κs) remain stable for a shortened version from the beginning but are significantly higher for a shortened version from the middle of the two situations. They are significantly lower, if the shortened version is taken from the end of the crafting and free-play situation. Regarding *Joint Attention*, observer-agreement indices (i.e., Cohen κs) of shortened versions from the beginning or the middle are significantly lower than those of the full-length videos, whilst observer-agreement indices of shortened versions from the end of the two situations are significantly higher than those of the full-length videos. Results from analyses of inter-rater agreement support the possibility of coding only segments from the beginning or the middle of the two situations. Those sequences exhibit observer-agreement indices that are either not significantly different from or even higher than those of the full-length videos (*Sensitivity* and *Feedback*) or significantly lower but still at a substantial level (*Joint Attention*), according to commonly used benchmarks (Landis and Koch 1977). Our results indicate that sequences from the end should be treated with caution, especially concerning *Feedback*, for which observer-agreement indices drop to only moderate levels.

Regarding maternal *Sensitivity* and maternal *Feedback*, there were no differences in results when coding only 4 min of the crafting situation and 6 min of the free-play situation as compared to the remaining parts of the videos for all three time points. However, regarding *Joint Attention*, this was only the case when the shortened sequence was taken from the middle of the two situations. Specifically, when the sequence was taken from the beginning, results differed from results from the remaining parts of the videos. Results from the comparison between the shortened versions and the remaining parts of the videos advise coding shortened sequences taken from the middle of the two situations.

Most correlations between shortened sequences and the remaining parts of the videos were in the medium to large range. Therefore, we can conclude that mother-child dyads predominantly retain their relative position when short segments instead of long sequences are used. For those behavioral categories that exhibit low correlation values, our data show that most of them occur either not at all or very frequently in the respective situations (refer to Table 2). Particularly, *Feedback* is rarely used in free-play situations.

For all three INTAKT dimensions, differences between the crafting situation and the free-play situation appeared as expected. Because the crafting situation is a structured situation with a defined task that mother and child should accomplish together, it elicits a maternal interactive style that is characterized by more feedback to the child, more directions given to the child, and a somewhat lower sensitivity level. This behavior differs from the one that is prevalent in the free-play situation, which is characterized by a higher level of actively playing together. Those differences are in line with theory and previous research (e.g., Grusec and Davidov 2010; Mateus et al. 2013). For all three time points, the shortened versions appear to validly represent the differences between the crafting and the free-play situation.

In conclusion, our study shows that it is possible to reliably and validly assess the quality of maternal interactive behavior via the behavioral observation method INTAKT, even when only coding 10 min of an interaction. In all, selecting slices from the middle of interactions seems preferable, because results from these sequences seem to most closely match results from full-length interactions.

Some limitations of this study should be noted, though. First, reliability was only assessed in terms of inter-rater agreement, but stability over time was not assessed. Future investigators may wish to assess test-retest reliabilities, thus providing further insights into the reliability of INTAKT and possible temporal effects. Second, all videos used for this study were taken from different stages during the development of the INTAKT measure. As is common in the development of behavioral-observation tools, category definitions and coder instructions become refined over time, which should lead to an improvement of inter-rater reliability. Therefore, most averaged estimates for inter-rater reliability exhibit only moderate to good values. However, this means that our results can be considered to represent a lower threshold of reliability estimates.

Finally, videos that have been used in this study varied in length, which may be indicative of potentially moderating factors due to sample characteristics. For instance, mothers who have been spending a long time interacting with their children may have become tired and strained and may therefore have become less attentive or responsive towards their child. Consequently, this may have resulted in more negative observations in longer videos. In a different vein, mothers who do not experience much joy in interacting with their children may have cut the situation short, which could have yielded more negative observations in shorter videos. However, we aimed at reducing influences of such possible moderators by excluding untypically long and short videos (refer to Methods). Future researchers may wish to investigate the influence of total video length on the observed behaviors.

In conclusion, our findings show that thin slices are a viable option when coding behavioral-observation data. In accordance with the findings of James et al. (2012), we recommend investigating the ideal duration of slices for specific observational methods. However, we demonstrate that coding a middle slice of planned observations may provide a useful alternative to coding full-length observations in practical contexts, at least when using the INTAKT.
